# Clinical prospects for laparoscopic stoma closure of a temporary loop ileostomy: Initial experience and report

**DOI:** 10.1111/ases.12790

**Published:** 2020-02-17

**Authors:** Yoshiaki Kita, Shinichiro Mori, Kan Tanabe, Kenji Baba, Kiyonori Tanoue, Tetsuya Idichi, Masumi Wada, Takaaki Arigami, Ken Sasaki, Kosei Maemura, Shoji Natsugoe

**Affiliations:** ^1^ Department of Digestive Surgery, Breast and Thyroid Surgery Graduate School of Medicine, Kagoshima University Kagoshima Japan

**Keywords:** closure of stoma, ileostomy, laparoscopic surgery

## Abstract

**Introduction:**

In closure of a stoma, the small working space and adhesions hinder a precise surgical procedure, compared with conventional approaches to digestive surgery. The aim of this prospective study was to introduce a new technique of laparoscopic stoma closure (LASC).

**Materials and surgical techniques:**

After starting with three trocars, it is a priority to dissect around the arising ileum; a linear stapler is precisely inserted in both orifices of the loop stoma and applied two times, extracorporeally. Ultimately, both the oral and anal sides of the loop ileum are cut and closed using a linear cutter stapler in a delta‐shaped manner just under the abdominal wall, intracorporeally. Eventually, the arising stoma is removed using an intra‐abdominal and cutaneous approach.

**Discussion:**

LASC for patients with a temporary loop ileostomy is safe and feasible. More data and experience will be required to verify the benefits of this new technique.

## 1 INTRODUCTION

The frequency of creating a temporary diverting stoma is increasing with various digestive surgeries including radical resection of colorectal cancer and is used to stabilize the anastomosis and avoid serious consequences due to anastomotic leakage.^1^, ^2^ Therefore, the frequency of closing temporary diverting stomas is also increasing. Stoma closure is recognized as a relatively less invasive digestive surgery because fatal complications and morbidity associated with anastomotic leakage are rare.^3^, ^4^ On the other hand, several reports have investigated wound infection after stoma closure in line with anastomotic leakage and related deaths.^5^, ^6^, ^7^, ^8^

Focusing on the surgical procedure, the adhesions around the arising stoma and tight working space with a small skin incision always hinder us during the procedure, including dissection of the adhesion and resection of the stoma. Moreover, returning the anastomosis into the abdominal cavity sometimes is difficult, often resulting in postoperative ileus due to anastomotic edema. To improve the procedure and reduce these interferences, here we developed a new technique of laparoscopic stoma closure (LASC) and accompanied short outcome. The technique used in this pilot study has the potential to contribute to patient wellbeing when closing a stoma.

## 2 MATERIALS AND SURGICAL TECHNIQUES

All patients in the present study underwent a diverting ileostomy with laparoscopic rectal resection except for two whole colon laparoscopic resections for inflammatory bowel disease, and all stomas were located in the terminal ileum. This pilot study was approved by our institutional review board (approval No. 27‐228) and all enrolled patients consented to their participation after sufficient explanation of this study.

All patients underwent general anesthesia and were placed in a supine position.

**Figure 1:**
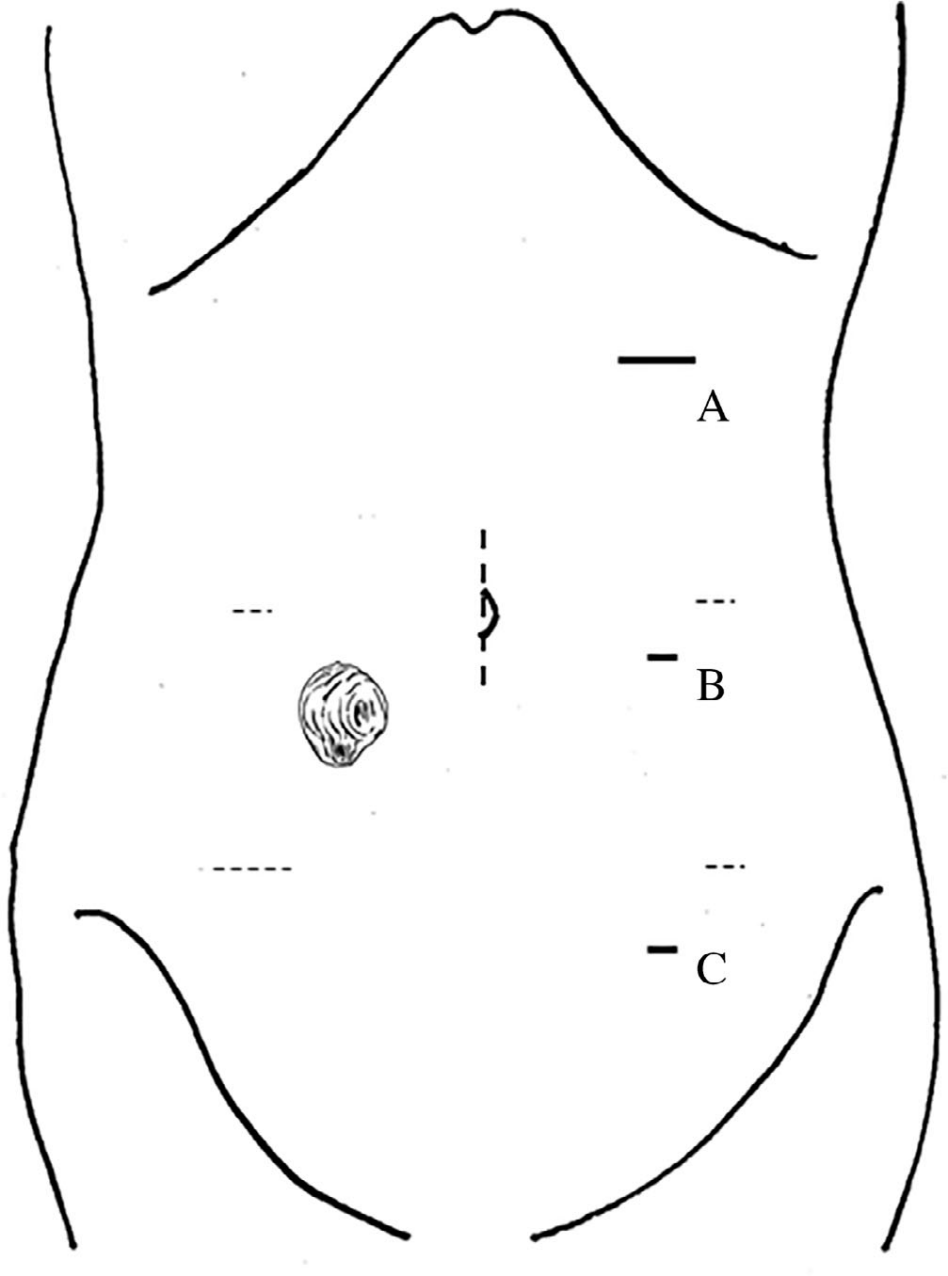
Skin incisions for trocar placement. Dashed lines are simulated incisions in previous laparoscopic operation. (A) 12 mm trocar with upper abdomen apart from previous incision. (B) 5 mm trocar in the left mid abdomen. (C) 5 mm trocar in the left low abdomen

**Figure 2:**
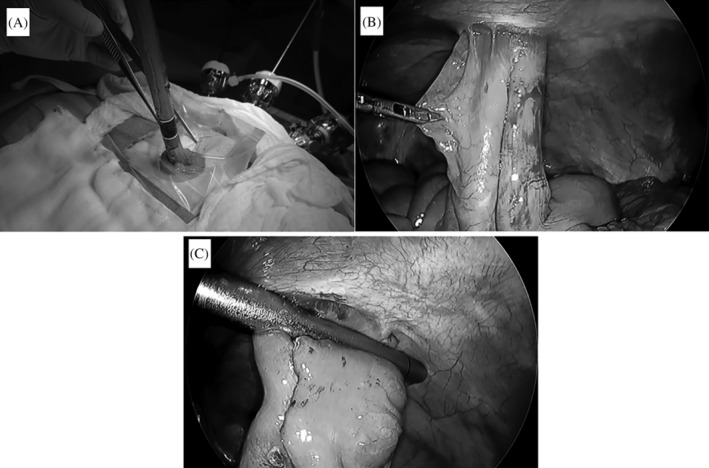
A, Insertion of a liner stapler though the orifices of a loop ileostomy. B, Inside view of the insertion and application of a liner stapler. C, Detachment by a liner stapler just under the abdominal wall from the 12‐mm trocar after anastomosis

After a 12‐mm trocar, a 5‐mm flexible scope was inserted keeping distance using an open method. Pneumoperitoneum was maintained at 12 mm Hg with carbon dioxide, and two 5‐mm trocars were placed under laparoscopic guidance (Figure 1). It was a priority to precisely dissect intracorporeally the adhesion arising around the ileum. Then, a linear stapler was gently inserted in both orifices of the loop stoma gently extracorporeally (Figure 2A) and applied two times for sufficient anastomosis while keeping straight the ileum inside the abdominal cavity with laparoscopic assistance and observation (Figure 2B). Both the oral and anal sides of the loop ileum were cut and closed intracorporeally using a linear cutter stapler in a delta‐shaped manner just under the abdominal wall (Figure 2C). It is necessary to avoid triple overlap of staple lines and the residual mesothelium was treated with an appropriate surgical device. The overlap of two staple lines was not reinforced, but was covered using the greater omentum. Ultimately, the extracting stoma and remaining small intestine are removed with cutaneous approach after exposure of the abdominal rectus muscle with laparoscopic dissection. The skin suture is a circumferential subcuticular wound approximation or conventional simple skin suture after the stoma closure.

## 3 DISCUSSION

The development of surgical procedures, for instance, very low anterior resection including intersphincteric resection increases the frequency of temporary stomas.^9^ According to a randomized, controlled study, ileostomy with low anterior resection has definite advantages in terms of mortality and recurrence and reduces risks related to surgery compared with low anterior resection without ileostomy, which may result in anastomotic leakage, postoperative ileus, slow resumption of diet, wound infection, small bowel obstruction.^10^

Moreover, more recently, it is now conventional to conduct entirely laparoscopic procedures, and laparoscopic diverting ileostomy has been widespread and reported as a useful surgical procedure for low anterior resection of rectal cancer.^11^, ^12^ Although the closure of a colostomy or ileostomy requires less invasive surgery in general than the initial surgery, postoperative complications occur in 24.6% to 48% of cases overall.^13^, ^14^ Moreover, combining the closure of stoma in patients with risk factors including diabetes and obesity compels us to consider how we might mitigate complications as far as possible.

In ordinary closure of the stoma, the adhesion should be released from subcutaneous tissue and the abdominal wall, which is the initial procedure, but it is difficult to approach this procedure using a small skin incision and viewing field. Blind dissection is likely to lead to postoperative complications. Moreover, it is definitely necessary to remove and raise the ileum for anastomosis including ileostomy for sufficient safety when using a linear stapler.^15^, ^16^

Here, we report a new technique to conduct complete LASC for ileostomy. It was possible to observe around and under the stoma inside the abdominal cavity in a safe manner. Moreover, the anastomosis using a linear stapler without treating the mesothelium inside the abdominal cavity allows minimal manipulation in this procedure. Loop ileostomy closure after laparoscopic colorectal surgery was reported to have a relatively shorter operative time and hospital stay and a lower rate of postoperative complications than after open procedure.^17^ On the other hand, this closure with a complete laparoscopic technique is the first report worldwide to our knowledge, and reinforces the advantages of laparoscopic surgery.

This pilot study compared and evaluated complete LASC with the conventional approach. This new method is not inferior to the conventional approach. Nine patients from January 2015 to April 2018 were enrolled for this prospective study and underwent LASC. The mean operative time was 129 minutes and blood loss was 10 g. No patient had intraoperative complications. Two patients (22.2%) had postoperative complications. One patient had a surgical site infection and recovered after conservative treatment. One patient developed postoperative anastomotic bleeding, which required endoscopic intervention, and then recovered after treatments. None of the patients had unplanned readmissions into hospital within 30 days. There was no mortality. Meal intake was 4.6 days and the length of hospital stay was 11 days after surgery. In addition, no significant difference was found for each clinical parameter including body mass index and short‐term outcome compared with a conventional approach in the same terms (Tables S1 and S2). This new method is not inferior to the conventional approach. This study has some limitations. It may have been subject to selection bias because this was a single‐center study with a small population. Although this new technique should overcome some issues and become widely and usually used, ventral hernia around the stoma and adhesion with mesothelium on the opposite site from the surgical manipulating direction may hinder this new procedure. Therefore, indications for this surgical procedure should be defined with precise pre‐operative information and consideration under pre‐operative diagnosis. It is necessary to accumulate more cases and experience to provide substantial evidence for this new surgical procedure.

In conclusion, we have had success with and report herein for the first time to our knowledge, complete LASC of a temporary loop ileostomy for selected patients. This technique allows us to avoid blind and rough dissection under a small view and working space during open stoma closure. This is a report of a small pilot study and it is necessary to accumulate cases and experiences. Standardization of this new technique in further studies has the potential to reduce operation time and postoperative complications, and to improve prognosis.

## CONFLICT OF INTEREST

The authors have no conflict of interest in relation to the article.

## ETHICS STATEMENT

This study was approved by our institutional review board in Kagoshima University Hospital (approval No. 27‐228) and all enrolled patients consented to their participation after sufficient explanation of this study.

## Supporting information


**Supplemental Table S1**. Patients and pre‐operative characteristics with nine patients in this study and conventional approach

**Supplemental Table S2**. Comparison between laparoscopic and conventional group
