# Supplementary figures and images for: Clinical prospects for laparoscopic stoma closure of a temporary loop ileostomy: Initial experience and report
Source: Asian J Endosc Surg. 2020 Feb 17;13(4):618–21. doi: 10.1111/ases.12790 (PMC7687255; doi:10.1111/ases.12790)

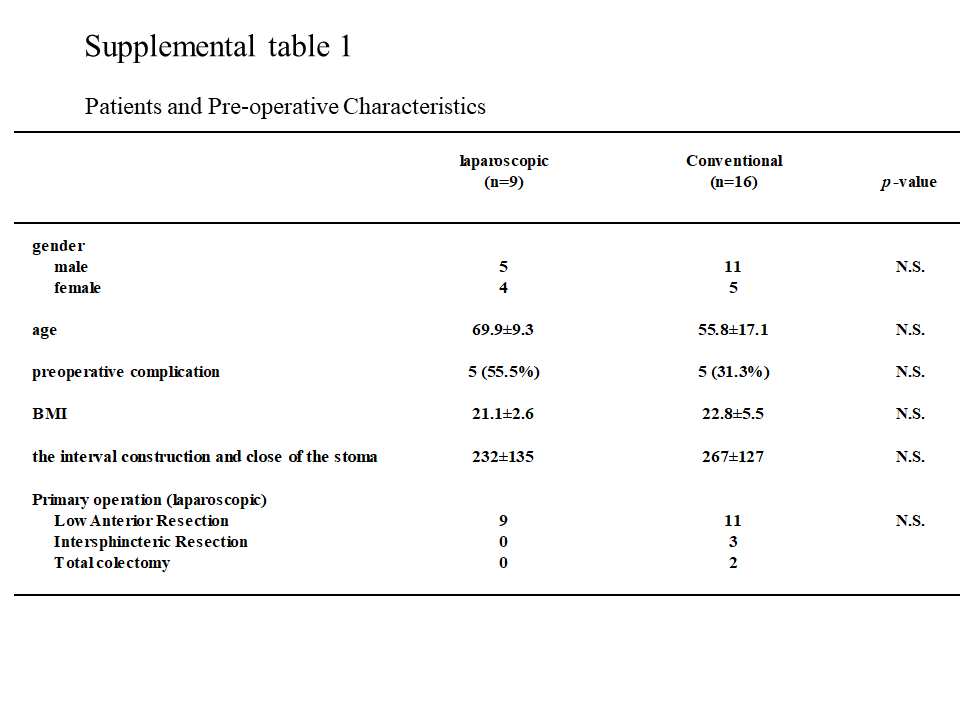

Supplement: Supplementary file 1 — Supplemental Table S1. Patients and pre‐operative characteristics with nine patients in this study and conventional approach [file ASES-13-618-s001.tif]

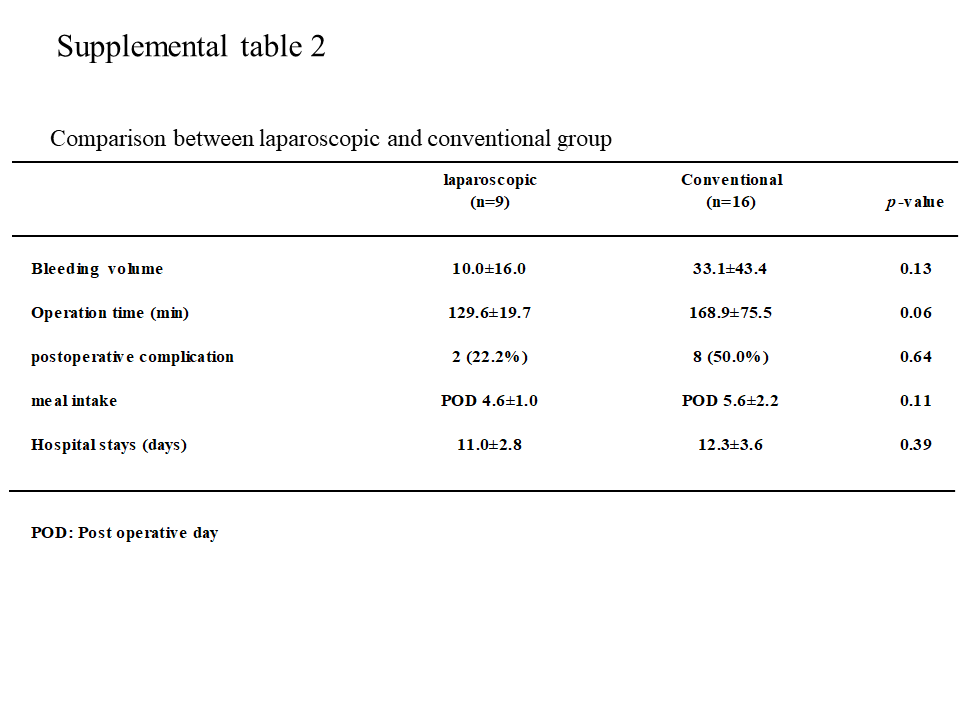

Supplement: Supplementary file 2 — Supplemental Table S2. Comparison between laparoscopic and conventional group [file ASES-13-618-s002.tif]
